# Association between frequent use of nonsteroidal anti-inflammatory drugs and breast cancer

**DOI:** 10.1186/1471-2407-5-159

**Published:** 2005-12-12

**Authors:** Elham Rahme, Joumana Ghosn, Kaberi Dasgupta, Raghu Rajan, Marie Hudson

**Affiliations:** 1 Division of Clinical Epidemiology, McGill University Health Centre Research Institute, Montreal, Canada; 2 Division of Medical Oncology, McGill University Health Center, Montreal, Canada

## Abstract

**Background:**

Eighty percent of all breast cancers and almost 90% of breast cancer deaths occur among post-menopausal women. We used a nested case control design to examine the association between nonsteroidal anti-inflammatory drug (NSAID) use and breast cancer occurrence among women over 65 years of age. The cyclooxygenase (COX)-2 enzyme is expressed more in breast cancers than in normal breast tissue. COX-2 inhibition may have a role in breast cancer prevention.

**Methods:**

In the Canadian province of Quebec, physician services are covered through a governmental insurance plan. Medication costs are covered for those ≥ 65 years of age and a publicly funded screening program for breast cancer targets all women 50 years of age or older. We obtained encrypted data from these insurance databases on all women ≥ 65 years of age who filled a prescription for COX-2 inhibitors, non-selective NSAIDs (ns-NSAIDs), aspirin, or acetaminophen between January 1998 and December 2002. Cases were defined as those women who have undergone mammography between April 2001 and June 2002 and had a diagnosis of breast cancer within six months following mammography. Controls included those who have undergone mammography between April 2001 and June 2002 without a diagnosis of any cancer during the six months following mammography. The exposure of interest, frequent NSAID use, was defined as use of ns-NSAIDs and/or COX-2 inhibitors for ≥ 90 days during the year prior to mammography. Frequent use served as a convenient proxy for long term chronic use.

**Results:**

We identified 1,090 cases and 44,990 controls. Cases were older and more likely to have breast cancer risk factors. Logistic regression models adjusting for potential confounders showed that frequent use of ns-NSAIDs and/or COX-2 inhibitors was associated with a lower risk of breast cancer (OR: 0.75, 95% confidence interval 0.64–0.89). Results were similar for COX-2 inhibitors (0.81, 0.68–0.97) and ns-NSAIDs (0.65, 0.43–0.99), when assessed separately. Frequent use of aspirin at doses > 100 mg/day in the year prior to mammography was also associated with a lower risk of breast cancer (0.75, 0.64–0.89). However, use of aspirin at doses ≤ 100 mg/day did not have any association with breast cancer (0.91, 0.71–1.16).

**Conclusion:**

Women who use NSAIDs or doses of aspirin > 100 mg frequently may have a lower risk of breast cancer.

## Background

Breast cancer is the most frequently diagnosed cancer and the second leading cause of cancer death in Canadian women[1]. Eighty percent of all breast cancers and almost 90% of breast cancer deaths occur among women 50 years of age or older [1]. Mammographic screening is an effective means of early breast cancer detection among women in this age group[2]. Studies have shown that aspirin and non-aspirin nonselective nonsteroidal anti-inflammatory drugs (ns-NSAIDs) may have a role in the prevention of breast cancer in the general population. [3-8] Ns-NSAIDs and the cyclo-oxygenase (COX)-2 inhibitors inhibit the COX-2 enzyme that is expressed more in breast cancers than in normal breast tissue[9][10] In vitro and animal studies have shown that the inhibition of COX-2 may prevent tumor formation and growth [11-15].

The well known serious gastrointestinal (GI) toxicity of ns-NSAIDs, thought to be due to the inhibition of the constitutive form of COX, the COX-1 enzyme, has prevented their use for chemoprevention. The COX-2 inhibitors have less GI toxicity compared to ns-NSAIDs [16,17]. These agents appeared to be an attractive option for cancer chemoprevention until recently when concerns about their cardiovascular (CV) safety were raised. In fact, in 2004, two cancer prevention trials [18-20] were terminated prematurely because patients randomized to the COX-2 inhibitor arms experienced more CV events compared to patients given placebo.

Continuous use of ns-NSAIDs or COX-2 inhibitors for cancer chemoprevention in healthy individuals appears problematic. However, patients who suffer from arthritis have few other options to control their pain and inflammation. Acetaminophen alone at regular doses may be inadequate for these patients and high doses are associated with hepatotoxicity[21].

We sought to determine whether frequent use of NSAID or aspirin reduces the risk for breast cancer. Although ns-NSAIDs and aspirin may be associated with GI toxicity and COX-2 inhibitors may increase CV risk, among patients who require NSAIDs for pain control or aspirin for cardioprotection, breast cancer risk reduction may be an incidental benefit.

## Methods

### Data source

The Québec provincial government health insurance agency, the Régie de l'Assurance Maladie du Québec (RAMQ), covers all in-patient and out-patient medical services for the entire population of the Canadian province of Québec including a screening program for breast cancer offered to all women 50 years of age or over. The RAMQ also covers the costs of outpatient prescription drugs for all individuals aged 65 years and older residing in Quebec. RAMQ has provided unrestricted coverage for ns-NSAIDs, aspirin and acetaminophen for at least the last two decades, celecoxib since October 1999, and rofecoxib since April 2000 until its withdrawal in September 2004. Ibuprofen, aspirin and acetaminophen are also available over-the-counter. The data in the drug claims database have been validated for accuracy of prescription claims[22] and have been used in other studies [23-25].

### Source population

From the RAMQ database, we obtained in- and out-patient physician and procedure claims, prescription drug claims, and demographic data on all patients aged 65 years and older who were dispensed COX-2 inhibitors, ns-NSAIDs, aspirin, or acetaminophen between January 1998 and December 2002 in the province. Patients retained from this population were 1) female, 2) ≥ 66 years of age and therefore had been covered by the drug plan for at least one year, and 3) had undergone bilateral mammography between April 2001 and June 2002. The date of the bilateral mammography was defined as the index date. Women who, in the three years prior to the index date, had a diagnosis of any type of cancer, or a mastectomy were excluded. Under the assumption that women undergoing mammography for screening purposes require these tests at no more than yearly intervals, we excluded patients who underwent a mammogram in the year prior to the index date. RAMQ uses the same diagnostic codes as those given by the *International Classification of Diseases*, 9^th ^review (ICD-9). RAMQ procedural codes are available upon request.

### Study design and selection of cases and controls

We conducted a nested case-control study. From the source population defined above, we identified cases and controls. The cases were those women who underwent a diagnostic or therapeutic test or procedure of the breast (fine needle aspirate, puncture, core or excision biopsy, capsulectomy or mastectomy), and who were given a diagnosis of breast cancer (ICD codes 174.0 to 174.9 and 233.0) during the six months following the index date. The controls were those patients who were not diagnosed with breast cancer or any other type of cancer in the six months following the index date.

### Exposure

We considered regular use of pain suppressing medications in the year prior to the index date as an indicator of chronic use of these medications in preceding years. Patients who filled a prescription for any ns-NSAID, COX-2 inhibitor, aspirin or acetaminophen in the year prior to the index date were identified. The number of days supplied for each study drug during that year was recorded.

The primary exposure was the filling of one or more prescriptions for celecoxib and/or rofecoxib and/or any ns-NSAID for a total of ≥ 90 days in the year prior to the index date. We also identified patients who were exposed to ≥ 90 days of aspirin and patients exposed to ≥ 90 days of acetaminophen in the year prior to the index date. Subgroups of exposure were defined for those exposed to ≥ 90 days of ns-NSAIDs, both COX-2 inhibitors, and rofecoxib and celecoxib, separately. We also separated exposure to ≥ 90 days of aspirin into two groups according to the mean daily dose prescribed, whether ≤ 100 mg or >100 mg. Non-exposed patients were those who did not receive any prescription for a COX-2 inhibitor, ns-NSAID, aspirin or acetaminophen in the year prior to the index date.

### Patient characteristics at the index date

The baseline patient characteristics available from the RAMQ database were determined based on demographic data, prescription claims, and in- and out-patient physician and procedure claims. They were: 1) age, defined categorically as 66–74, 75–84 and ≥ 85 years old; 2) a history of diagnostic and therapeutic breast tests and procedures, namely fine needle aspirate, puncture, core or excision biopsy or capsulectomy, in the three years prior to the index date; 3) a diagnosis of benign neoplasm of the breast (ICD code 217) in the three years prior to the index date; 4) mammography in years 2 or 3 prior to the index date; 5) a diagnosis of another breast disease in the three years prior to the index date (ICD codes 238.3, 239.3, 793.8 and 610.0 to 611.9); 6) estrogen replacement therapy in the year prior to the index date; 7) combined estrogen and progesterone replacement therapy in the year prior to the index date; and 8) visit to a gynecologist in the year prior to the index date.

### Statistical analysis

The baseline patient characteristics of the cases and controls were compared using descriptive statistics.

#### Primary analysis

Logistic regression models were used to determine the association between exposure to ≥ 90 days of ns-NSAIDs and/or COX-2 inhibitors, aspirin at doses ≤ 100 mg/day, aspirin at doses >100 mg/day and acetaminophen and breast cancer, using the non-exposed patients as the reference group. The models were adjusted for patient characteristics at the index date, as defined above. We adjusted for whether patients had at least one visit to a gynecologist in the year prior to the index date and whether they had mammography in the years 2–3 prior to the index date to reduce a possible surveillance bias.

#### Secondary analysis

We conducted an analysis identical to the primary analysis including only patients who also underwent mammography in the years 2–3 prior to the index date. This limited the analysis to those women who were adherent to the screening program. We also examined the effect of exposure to at least 90 days of ns-NSAIDs and/or COX-2 inhibitors excluding the prescriptions filled in the month prior to index date. This analysis would exclude prescriptions that could have been prescribed for symptoms related to the referral of the patient to mammography.

#### Sample size calculation

We calculated that 558 cases and 5578 controls (a total sample size of 6136) were needed for a two-group continuity corrected chi-square test with a 0.05 two-sided significance level to have 80% power to detect an odds ratio of 0.72 between the cases and the controls, assuming that 15% of cases would be exposed. This sample size calculation was based on a ratio of 10 controls to 1 case. However, since matching on all or some of the patient characteristics may introduce biases and, more importantly, since there were no costs associated to the selection of controls, we decided to use all eligible controls for the analysis.

All analyses were performed using the statistical software SAS version 8.2 (SAS Institute Inc., Cary, North Carolina). We performed our sample size calculation using nQuery20.

## Results

### Study population

Among 418,458 women in our dataset, 68,641 were at least 66 years of age and had undergone bilateral mammography between April 2001 and June 2002. Of these patients, 46,080 fulfilled eligibility criteria, including 1,090 (2.4%) cases and 44,990 (97.6%) controls. Patient characteristics are presented in Table 1. Patients diagnosed with breast cancer were slightly older than controls. Compared to controls, cases more commonly had a history of diagnostic and therapeutic breast procedures and a history of benign neoplasm of the breast or other breast diseases in the three years prior to the index date. Cases used estrogen replacement therapy less frequently than controls and were less likely than controls to have seen a gynecologist in the year prior to the index date. Twenty one cases (1.9%) and 101 controls (0.2%) died during the 6-month period following the index date.

### Exposure to the drugs of interest

Frequency of exposure to the medications of interest are indicated in Table 2 (≥ 90 days of ns-NSAIDs/COX-2 inhibitors, aspirin, acetaminophen). Controls were more likely than cases to be exposed to ≥ 90 days of ns-NSAIDs and to COX-2 inhibitors. Patients exposed to at least 90 days of either ns-NSAIDs or COX-2 inhibitors during the year prior to the index date filled on average (± standard deviation) 8.0 ± 4.4 prescriptions for these drugs during that year with a median number of 7 prescriptions. Ninety percent of all aspirin prescriptions were for 325 mg/day or less and 25% of them were for ≤ 80 mg/day, the median dose (interquartile range) for all aspirin prescriptions were 312.5 (80, 312.5).

### Patient characteristics associated with breast cancer

Logistic regression models showed that cases were older (odds ratio, 95% confidence interval for 75–84 years and ≥ 85 years compared to 66–74 years: 1.90, 1.66–2.18 and 3.68, 2.69–5.04, respectively) and more likely to have received combined estrogen therapy 1.35 (1.06, 1.70) in the year prior to the index date. Cases were also more likely to have a history of diagnostic/therapeutic breast procedures in the three years prior to the index date (2.60, 1.59–4.26), benign breast neoplasm diagnosed in the year prior to the index date (1.40, 1.12–1.75), and/or other breast disease in the three years prior to the index date (1.87, 1.57–2.23). Cases were less likely to have used estrogen replacement therapy in the year prior to the index date (0.71, 0.61–0.84). Cases were also less likely to have seen a gynecologist in the year prior to the index date (0.68, 0.57–0.81) and to have had mammography in the years 2–3 prior to the index date (0.29, 0.26–0.33) (data not shown).

Table 2 shows the crude and adjusted odds ratios of the association between exposure to the drugs of interest and breast cancer. Small differences between the crude and adjusted odds ratios were observed. We investigated these differences and found that they resulted from the adjustment for other exposure categories. In fact, exposure categories were not mutually exclusive. For example, some patients exposed to at least 90 days of ns-NSAIDs and/or COX-2 inhibitors were also exposed to at least 90 days of aspirin. When only exposure categories were entered in the model, the effect of each was adjusted for the effects of the others. None of the other patient characteristics listed above was a confounder or effect modifier for exposure to ns-NSAIDs and/or COX-2 inhibitor. However, age was a confounder for exposure to aspirin.

As shown in Table 2, exposure to at least 90 days of an ns-NSAID and/or COX-2 inhibitor (0.75, 0.64–0.89) was inversely associated with the risk of breast cancer as was exposure to at least 90 days of aspirin at an average daily dose > 100 mg (0.75, 0.64 – 0.89). In contrast, exposure to at least 90 days of aspirin at an average daily dose ≤ 100 mg and exposure to at least 90 days of acetaminophen were not found to be associated with breast cancer. Exposures to at least 90 days of COX-2 inhibitors (0.81, 0.68–0.97) or ns-NSAIDs (0.65, 0.43–0.99), assessed separately, were associated with a lower risk of breast cancer. Exposure to at least 90 days of celecoxib (0.88, 0.70–1.10) or rofecoxib (0.80, 0.63–1.02) showed trends toward an association with a lower risk of breast cancer.

### Secondary analyses

Consistent with the results of the primary analysis, exposure to ≥ 90 days of ns-NSAIDs and/or COX-2 inhibitors was also associated with a lower risk of breast cancer in a subgroup analysis of patients who had mammography in years 2–3 prior to the index date (0.73, 0.55–0.93). Again, in this subgroup, exposure to ≥ 90 days of acetaminophen (0.86, 0.59–1.31) and exposure to ≥ 90 days of aspirin at an average daily dose ≤ 100 mg (0.97, 0.68–1.39) were not associated with breast cancer. In contrast with the findings of the primary analysis, exposure to ≥ 90 days of aspirin at an average daily dose >100 mg (0.93, 0.72–1.18) did not seem to be associated with breast cancer in this subgroup. It is difficult to conclude if this difference was due to chance given the multiple modeling conducted in this study or to differences in the characteristics of the women in the two analyses. In particular, in the primary analysis, only 25% (54/211) of the cases who received aspirin for 90 days or more at an average daily dose >100 mg also received hormone replacement therapy compared to 42% (45/93) of women in the secondary analysis. Thus, there may have been better surveillance of the women in the secondary analysis. Of note, the effect of aspirin at an average daily dose >100 mg in the subgroup analysis differed between women who were not receiving hormone replacement therapy (0.83, 0.60, 1.15) and those who did (1.08, 0.74, 1.56).

The results of the main analysis did not change substantially when we reassessed the effect of exposure to ≥ 90 days of ns-NSAIDs and/or COX-2 inhibitors (0.75, 0.63–0.89), aspirin at an average daily dose ≤ 100 mg (0.90, 0.71, 1.15) aspirin at an average daily dose > 100 mg (0.75, 0.63, 0.89) and acetaminophen (0.89, 0.69, 1.15) excluding the prescriptions filled in the month prior to the index date.

## Discussion

In this population-based study of women 65 years of age or older, we found that patients taking ns-NSAIDs and/or COX-2 inhibitors and patients taking aspirin at doses >100 mg/day for a period of ≥ 90 days were at a lower risk of breast cancer compared to those not taking these medications. Given the cardiovascular risk associated with use of some COX-2 inhibitors and ns-NSAIDs, regular use of these agents for primary cancer chemoprevention cannot be recommended. However, for women who require these agents for pain control, a concurrent benefit of such treatment may be reduced risk of breast cancer. Aspirin is used most commonly for the prevention of myocardial infarction and stroke. Although aspirin doses of as little as 80 mg per day are effective for the prevention of cardiovascular events, our results suggest that higher doses may additionally protect women from developing breast cancer.

Ns-NSAIDs are COX-1 and COX-2 inhibitors. A significant inverse association has been observed in most observational studies between the use of ns-NSAIDs and the risk of breast cancer. In a meta-analysis of six cohort and 8 case-control studies, the authors found a significant 18% reduction in breast cancer risk among patients taking ns-NSAIDs compared to those not taking them. However, the data were insufficient to estimate a dose-response effect for duration and frequency of use of any particular type of ns-NSAID [3]. A subsequent case-control study found a 24% reduction in breast cancer risk with the regular use of any ns-NSAID for > 2 months and a stronger association in prolonged users compared to non-users (relative risk reduction of 32%) in those having used ns-NSAIDs for > 9 years compared to non-users[4]. A protective effect of ns-NSAIDs was recently confirmed in the Women's Health Initiative (WHI) where regular ns-NSAID use, defined as two or more tablets per week for 10 or more years, produced a 28% reduction in the risk of breast cancer[5]. A protective effect of ns-NSAIDs was also found in a case-control study where occasional as well as regular use of either prescription or non-prescription ns-NSAIDs were found to be inversely associated with invasive breast cancer as well as with carcinoma in situ of the breast [6]. More recently, a case control study found that long term regular use of ns-NSAIDs was associated with a decreased risk of breast cancer [7]. There is now growing evidence that the selective COX-2 inhibitor celecoxib protects against the development of breast cancer. Treatment with celecoxib has been shown to significantly reduce the risk of developing breast cancer in animal models[15], including a mouse model for HER-2/neu-induced breast cancer[26], in a dose-dependent fashion[27]. There have been no clinical studies published to date confirming these results in humans.

### Aspirin

Aspirin is also an irreversible COX enzyme inhibitor[10]. The literature is unclear as to whether aspirin protects against breast cancer, with some studies suggesting that it does and others suggesting that it does not[3,28]. We observed a protective effect on breast cancer of aspirin prescribed at an average dose >100 mg/day but lower doses of aspirin (≤ 100 mg/day) did not seem to have an effect. Whereas ns-NSAIDs are usually used in anti-inflammatory doses, aspirin is often used in low, anti-platelet doses (≤ 325 mg)[29]. However, the dose of aspirin necessary to have an anti-inflammatory effect is up to 100 times greater than the anti-platelet dose[10]. In fact, results from the Women's Health Initiative suggest that the regular use of low dose aspirin, defined as ≤ 100 mg per day, had no relationship with the incidence of breast cancer[28]. Similarly, an analysis of women in the Cancer Prevention Study II Nutrition cohort did not find any association between use of ns-NSAID or aspirin and breast cancer [30]. In contrast, a case-control study using hospital controls reported an inverse association between both regular and occasional use of aspirin and breast cancer [8]. Another case-control study using the General Practice Research database also showed a reduced risk of breast cancer associated with aspirin and acetaminophen use [31]. A third case-control study showed a reduction in risk of breast cancer with aspirin use among women with hormone receptor-positive tumors but not among women with hormone receptor-negative tumors [32]. Finally, a study using a cohort of postmenopausal women also showed a reduction in the risk of breast cancer associated with frequent aspirin use [33].

We cannot exclude the possibility, however, that the apparent protective effect of aspirin observed in our study was a result of surveillance bias among aspirin users. Women prescribed aspirin for prevention of cardiovascular disease may be more likely to consistently undergo preventive interventions such as mammography. Aspirin users may have therefore been more likely to have undergone regular mammographic screening prior to the age of 65 years. Those with a previous history of breast cancer so detected would have been excluded from our cohort. Consistent with this possibility, we found that the protective effect of aspirin prescribed at an average dose >100 mg/day did not persist when we restricted the analysis to women who have undergone mammography in years 2–3 prior to the index mammogram.

### Limitations

In this study, prescription data were available starting in 1998. However, we used only one year of data to assess frequent use of the study drugs because in Quebec, patients are covered by the provincial drug plan starting at their 65^th ^birthday. Therefore, to have one full year of prescription data available at index date, women had to be 66 years of age or older at that date. To correctly examine exposure to the drugs of interest in the past 3 years, for example, women would have had to be at least 68 years of age at the index date. In designing the study, we thus had to trade-off the number of patients with the duration of exposure studied.

Patients may have used over-the-counter aspirin or ibuprofen (in contrast, rofecoxib, celecoxib and the other ns-NSAIDs are not available over-the counter in Québec). A survey conducted by Santé Québec, a provincial public health agency, showed that in 1998, 17.0% and 2.2% of the elderly who consumed ns-NSAIDs or aspirin, respectively, acquired them over-the-counter[23]. Québec has maintained the same drug insurance policy since 1997. The high number of patients who acquired ns-NSAIDs over-the-counter suggests that some patients may have been misclassified according to exposure status and duration of use. Therefore, some patients in the non-exposed group could have been exposed to ns-NSAIDs or aspirin which may have biased the results towards the null and could have made it more difficult to find an association. Thus, our estimate of the protective effect of ns-NSAIDs may be an underestimate.

A number of variables associated with breast cancer risk are not available in the RAMQ database, including family history of breast or other cancers, carriage of certain genes and gynecologic and obstetric history. However, these variables are not likely to be associated with exposure to ns-NSAIDs or COX-2 inhibitors and thus are unlikely to have confounded the results. Obesity, however, is a risk factor for breast cancer in post-menopausal women[34] as well as for osteoarthritis[35]. As such, it may be associated with both the exposure, namely ns-NSAIDs and/or COX-2 inhibitors, and the outcome and could be a potential confounder. However, if we postulate that cases were more likely to be obese and therefore more likely have osteoarthritis, then they would have used more ns-NSAIDs or COX-2 inhibitors than controls and this would have biased our results towards the null. Thus, again, our results would then underestimate the magnitude of the true association.

We are aware that the main exposure of at least 90 days of ns-NSAIDs and/or COX-2 inhibitors may seem to be a relatively short period of time to attribute a protective effect to a drug on a malignancy that may evolve over many years. However, existing literature also suggests that relatively short durations of exposures to cyclooxygenase inhibitors may protect against the progression of tumors. Recent randomized trials in the field of colorectal adenomas have shown that exposure to aspirin significantly reduced the number and histological grade of recurrent adenomas[36,37]. This difference is seen as early as three to six months after randomization[36].

Moreover, we believe that a minimum of 90 days of exposure is a marker for chronic exposure to ns-NSAIDs in the past. In fact, among women for whom 3 years of data were available prior to their index date and who used ns-NSAIDs and/or COX-2 inhibitors for at least 90 days in the year prior to the index date, 91% had also used them in years 2–3 prior.

## Conclusion

The ideal agents for chemoprevention and chemotherapy are those that are effective but that have few side effects. Although both ns-NSAIDs and COX-2 inhibitors seem to have similar protective effects on breast cancer, a continuous use of ns-NSAIDs or COX-2 inhibitors for cancer chemoprevention in healthy individuals appears problematic. However, in those women who have arthritis and are in need of frequent use of analgesics, ns-NSAID or COX-2 inhibitor use may offer protection against breast cancer compared with acetaminophen. Although we cannot exclude the possibility of surveillance bias among aspirin users, our findings support the possibility that at doses above 100 mg daily, aspirin may offer protection against the development of breast cancer. Many post-menopausal women require aspirin for the prevention of cardiovascular events. Increasing the dose from 80 mg daily to > 100 mg daily in these women may additionally provide protection against the development of breast cancer. This possibility has important public health implications and requires further investigation.

## Financial competing interests

Dr Rahme is a research scholar and Dr Hudson is a research fellow funded by The Arthritis Society. Drs Dasgupta and Rajan are physician scientists funded by the Fonds de la recherche en santé du Québec. This study was partially funded by the Arthritis Society.

Dr Rahme has served as consultant for Merck & Co. Inc. and Pfizer Inc., the companies that manufacture rofecoxib and celecoxib, in relation to other studies.

## Non-financial competing interests

The author(s) declare that they have no competing interests.

## Contributions of authors

All authors declare that they had full access to the data and participated in the design and conduct of the study, in the analysis and the interpretation of the data and in the writing of the report. All authors have seen and approved the final version. All authors had final responsibility for the decision to submit the paper to publication.

## Pre-publication history

The pre-publication history for this paper can be accessed here:



## Figures and Tables

**Table 1:** Patient characteristics at the index date and mortality in the following 6 months

	**Cases (%)**	**Controls (%)**
Number of patients	1,090	44,990
Mean age at index date ± SD	74.1 ± 5.7	71.1 ± 4.3
Number of patients 66–74	670 (61.5)	36,900 (82.0)
Number of patients 75–84	369 (33.9)	7,665 (17.0)
Number of patients ≥ 85	51 (0.05)	425 (0.9)
Risk factors		
Mammography in years 2 or 3 prior to the index date	514 (47.2)	35,229 (78.3)
History of diagnostic and therapeutic breast procedures in the 3 prior to the index date	19 (1.7)	296 (0.7)
Benign neoplasm of the breast in the 3 years prior to the index date	15 (1.4)	319 (0.7)
Other breast disease in the 3 years prior to the index date	169 (15.5)	4,667 (10.4)
Estrogen replacement therapy in the year prior to the index date	299 (27.4)	17,837 (39.7)
Combined estrogen/progesterone therapy in the year prior to the index date	101 (9.3)	4,943 (11.0)
Visit to a gynecologist in the year prior to the index date	152 (13.9)	10,076 (22.4)
Exposure to the drugs of interest (one or more prescriptions in the year prior to the index date)		
Ns-NSAID/COX-2 inhibitors	443 (40.6)	22,126 (49.2)
Ns-NSAIDs	102 (9.4)	5,572 (12.4)
COX-2 inhibitors	388 (35.8)	19,228 (42.7)
Rofecoxib	242 (22.2)	11,498 (25.6)
Celecoxib	203 (18.6)	10,269 (22.8)
Aspirin	329 (30.2)	13,391 (29.8)
Acetaminophen	253 (23.2)	10,159 (22.6)
Not exposed*	375 (34.4)	12,260 (27.3)

SD – standard deviation

*Not exposed refers to the group of patients who were not exposed to ns-NSAIDs, aspirin, COX-2 inhibitors or acetaminophen during the year prior to the index date

**Table 2:** Exposures to ≥ 90 days of COX-2 inhibitors, ns-NSAIDs, aspirin and acetaminophen in the year prior to the index date: Crude and adjusted odds ratios.

Exposure to ≥ 90 days	Cases N of patients (%)	Controls N of patients (%)	Crude odds ratios	Adjusted odds ratios*
Ns-NSAID/COX-2 inhibitors	186 (17.1)	8,681 (19.3)	0.70 (0.59, 0.84)	0.75 (0.64, 0.89)
COX-2 inhibitors	166 (15.2)	7,412 (16.5)	0.73 (0.61, 0.88)	0.81 (0.68, 0.97)
Rofecoxib	76 (7.0)	3,597 (8.0)	0.69 (0.54, 0.89)	0.80 (0.63, 1.02)
Celecoxib	93 (8.5)	3,792 (8.4)	0.80 (0.64, 1.00)	0.88 (0.70, 1.10)
Ns-NSAIDs	24 (2.2)	1,278 (2.8)	0.61 (0.40, 0.93)	0.65 (0.43, 0.99)
Aspirin				
average daily dose ≤ 100 mg/day	78 (7.2)	2,894 (6.4)	0.88 (0.69, 1.13)	0.91 (0.71, 1.16)
average daily dose >100 mg/day^#^	211 (19.4)	8,419 (18.7)	0.82 (0.69, 0.97)	0.75 (0.64, 0.89)
Acetaminophen	74 (6.8)	2,534 (5.6)	0.95 (0.74, 1.23)	0.91 (0.71, 1.16)
Not exposed ^§^	375 (34.4)	12,260 (27.3)	1 (Reference)	1(Reference)

* Adjusted for age, mammography in years 2 or 3 prior to index date, breast procedure in the prior 3 years, benign neoplasm of the breast in the prior 3 years, other breast disease in the prior 3 years, estrogen replacement therapy in the prior year, and visit to a gynecologist in the prior year- these variables were risk factors for breast cancer but their inclusion in the model did not alter substantially the effect of exposure to the drugs of interest.

^#^90% of all aspirin prescriptions were for ≤ 325 mg/day

^§^Not exposed refers to the group of patients who were not exposed to ns-NSAIDs, aspirin, COX-2 inhibitors or acetaminophen during the year prior to the index date
